# Gender, Age, and Ethnic Differences in Ultrasound Predictors of Malignancy Risk in Thyroid Nodules with Indeterminate Low-Risk Cytology (TIR3A): A Systematic Literature Review

**DOI:** 10.3390/diagnostics16213625

**Published:** 2026-11-03

**Authors:** Sandro La Vignera, Rosita A. Condorelli

**Affiliations:** Department of Clinical and Experimental Medicine, University of Catania, Via S. Sofia 78, 95123 Catania, Italy; rosita.condorelli@unict.it

**Keywords:** thyroid nodules, TIR3A, Bethesda III, ultrasound, gender differences, age, ethnicity, malignancy risk, TIRADS, microcalcifications

## Abstract

**Background:** Thyroid nodules classified as TIR3A (Italian Reporting System) or Bethesda III (atypia of undetermined significance/follicular lesion of undetermined significance) represent a diagnostic challenge with malignancy rates of 10–30%. While ultrasound features guide risk stratification, the influence of patient demographics—particularly gender, age, and ethnicity—on ultrasound predictors of malignant transformation remains incompletely characterized. **Methods:** We conducted a systematic literature review of studies evaluating ultrasound features in TIR3A/Bethesda III thyroid nodules, with specific focus on gender differences (male vs. female), age-related patterns, and ethnic variations (Caucasian vs. non-Caucasian). We extracted data on malignancy rates, odds ratios, relative risks, and diagnostic performance metrics (sensitivity, specificity, positive predictive value, negative predictive value, area under the curve) for individual ultrasound alarm signs stratified by demographic variables. A total of 20 studies were included (17 retrospective cohort studies, 3 prospective cohort studies); findings were synthesized qualitatively. **Results:** Male sex was reported as a risk modifier in small cohorts (crude relative risk 2.3; independent predictor status in one multivariate analysis without a reported adjusted odds ratio), but larger series found no significant sex effect; adjusted estimates are unavailable, and the association should be considered inconsistent and of uncertain magnitude. Age below 55 years was associated with higher malignancy rates and more aggressive ultrasound features in heterogeneous single-centre observational data; however, this threshold has not been validated for ultrasound-based malignancy prediction across populations. Direct Caucasian versus non-Caucasian comparative data are absent; observed geographic variation cannot be attributed to ethnicity given multiple confounders including iodine intake and healthcare system differences. Microcalcifications (sensitivity 32.4–87%, specificity 75–99.2%) and irregular margins (sensitivity 70%, specificity 94.9–95%) remain the most robust ultrasound predictors across demographic subgroups. **Conclusions:** Gender and age show associations with malignancy risk in TIR3A nodules, but these associations are inconsistent across cohorts and should not be treated as validated independent predictors. Ethnic comparative data remain insufficient. Optimal risk stratification should integrate demographic variables with ultrasound features and repeat cytology to personalize management and reduce unnecessary surgery.

## 1. Introduction

Thyroid nodules are highly prevalent in the general population, with detection rates of 19–68% by high-resolution ultrasound [1]. Fine-needle aspiration (FNA) cytology remains the cornerstone of preoperative diagnosis, yet 15–30% of thyroid nodules yield indeterminate cytology results [2]. The Bethesda System for Reporting Thyroid Cytopathology classifies these as Category III (atypia of undetermined significance/follicular lesion of undetermined significance, AUS/FLUS), corresponding to TIR3A in the Italian Reporting System for Thyroid Cytology [3]. These nodules harbor malignancy rates of 10–30%, creating a clinical dilemma between conservative surveillance and surgical intervention [4,5,6].

**Figure 1:**
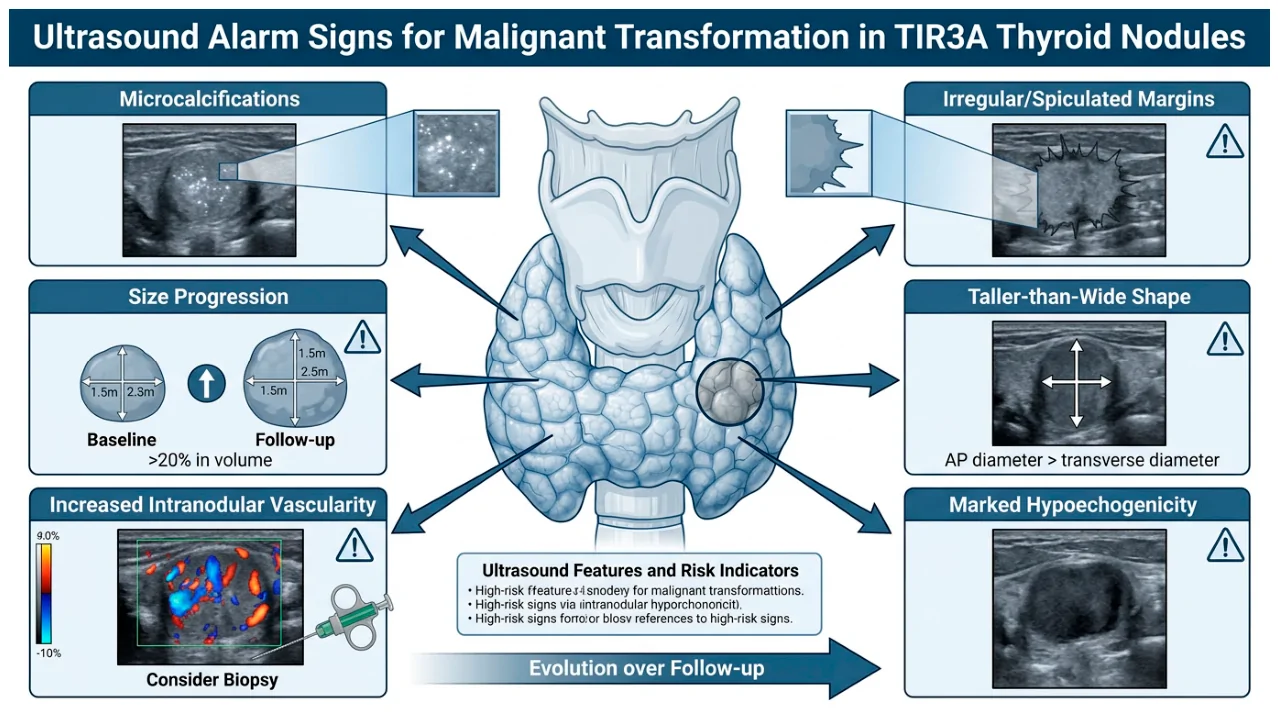
Ultrasound Alarm Signs for Malignant Transformation in TIR3A Thyroid Nodules (TIR3A/Bethesda III). The six primary alarm features are (1) **Microcalcifications**—punctate hyperechoic foci <1 mm without acoustic shadowing, representing psammoma bodies (sensitivity 32.4–87%, specificity 75–88.9%); (2) **Irregular/Spiculated Margins**—infiltrative borders with microlobulations or spiculations indicating extrathyroidal extension (sensitivity 70%, specificity 95–98.6%); (3) **Marked Hypoechogenicity**—echogenicity lower than surrounding strap muscles, suggesting high cellularity (sensitivity 79.2%, NPV 93.1%); (4) **Taller-than-Wide Shape**—anteroposterior diameter exceeding transverse diameter on transverse view, indicating centrifugal growth pattern (inconsistent predictive value in TIR3A); (5) **Increased Intranodular Vascularity**—prominent peri- and intralesional blood flow on color Doppler, associated with angiogenesis (*p* = 0.004 for malignancy association); (6) **Size Progression**—>20% increase in nodule volume or >2 mm growth in two dimensions during surveillance, serving as a trigger for repeat evaluation. The central thyroid anatomy illustrates bilateral lobes with multiple nodules demonstrating various alarm features. Evolution of these features over follow-up warrants repeat fine-needle aspiration or surgical referral. High-risk features (microcalcifications, irregular margins) are marked with warning symbols to emphasize their clinical significance.

Ultrasound (US) features have emerged as critical tools for risk stratification in indeterminate nodules. Multiple ultrasound risk stratification systems—including the American College of Radiology Thyroid Imaging Reporting and Data System (ACR TI-RADS), European Thyroid Association EU-TIRADS, and Korean TI-RADS—have been developed to standardize assessment [7,8,9]. Key ultrasound alarm features associated with malignancy include microcalcifications, irregular or spiculated margins, marked hypoechogenicity, taller-than-wide shape, and increased intranodular vascularity [10,11,12,13] (Figure 1; Table 1).

However, the predictive value of ultrasound features may be modulated by patient demographic characteristics. Emerging evidence suggests that gender, age, and ethnic background influence thyroid cancer epidemiology and biological behavior [19,20]. Female predominance in thyroid nodule prevalence contrasts with reports of higher malignancy rates in males with indeterminate cytology [21]. Age-related differences in tumor aggressiveness and ultrasound characteristics have been documented, with younger patients potentially exhibiting more aggressive features [22]. Ethnic and geographic variations in thyroid cancer incidence and molecular profiles are well established, yet their impact on ultrasound predictors in indeterminate nodules remains poorly characterized [23].

The primary objective of this systematic literature review is to investigate whether gender differences (male vs. female), specific age ranges, and ethnic background (Caucasian vs. non-Caucasian) modulate the probability of malignant ultrasound changes in TIR3A thyroid nodules, and to identify the combination of evolving ultrasound signs that best predict malignant transformation. By integrating demographic variables with ultrasound features, we aim to refine risk stratification algorithms and personalize management strategies for patients with indeterminate low-risk cytology. Specifically, the primary question is whether sex, age, and ethnic background modify the diagnostic performance of ultrasound features (sensitivity, specificity, and predictive values) for malignancy prediction in Bethesda III/TIR3A indeterminate thyroid nodules.

## 2. Methods

### 2.1. Literature Search Strategy

We conducted a comprehensive systematic literature search across three major electronic databases: PubMed/MEDLINE, Scopus, and Web of Science. The search was last run on 31 May 2026 and covered publications from January 2000 to May 2026. Database-specific search strings used controlled vocabulary (MeSH terms for PubMed/MEDLINE; Emtree for Scopus) combined with free-text terms in title and abstract fields; the complete syntax for each database is provided in Supplementary File S1. The initial search yielded 256 records; after deduplication, 198 unique records were screened. Three parallel search strategies were employed:

**Search 1 (Gender differences):** (“thyroid nodule” OR “thyroid cancer” OR “thyroid carcinoma”) AND (“TIR3A” OR “Bethesda III” OR “AUS/FLUS” OR “indeterminate cytology”) AND (“gender” OR “sex” OR “male” OR “female”) AND (“ultrasound” OR “ultrasonography” OR “TIRADS”) AND (“malignancy” OR “cancer risk”).

**Search 2 (Age-related patterns):** (“thyroid nodule” OR “thyroid cancer”) AND (“TIR3A” OR “Bethesda III” OR “indeterminate cytology”) AND (“age” OR “younger” OR “older” OR “age-related”) AND (“ultrasound” OR “ultrasonography”) AND (“malignancy risk” OR “transformation”).

**Search 3 (Ethnic differences):** (“thyroid nodule” OR “thyroid cancer”) AND (“TIR3A” OR “Bethesda III” OR “indeterminate cytology”) AND (“ethnic” OR “race” OR “Caucasian” OR “Hispanic” OR “African American” OR “Asian” OR “geographic variation”) AND (“ultrasound” OR “malignancy risk”).

These searches yielded 90 unique papers for gender analysis, 92 papers for age-related analysis, and 74 papers for ethnic/geographic variation analysis.

### 2.2. Inclusion and Exclusion Criteria

**Inclusion criteria:** -Studies evaluating thyroid nodules classified as TIR3A, Bethesda III, AUS/FLUS, or equivalent indeterminate low-risk cytology; -Studies reporting ultrasound features and their association with malignancy; -Studies providing demographic data (gender, age, ethnicity) with stratified analyses; -Studies with histopathological confirmation of malignancy; -Original research articles, prospective or retrospective cohort studies, and meta-analyses; -Publications in English.

**Exclusion criteria:** -Studies focusing exclusively on high-risk indeterminate cytology (TIR3B, Bethesda IV); -Pediatric populations (<18 years); -Case reports and case series with <20 patients; -Studies without histopathological confirmation; -Studies lacking ultrasound data or demographic stratification.

### 2.3. Data Extraction and Analysis

From each included study, we extracted: (1) study design and population characteristics; (2) cytological classification system; (3) demographic variables (gender distribution, age ranges, ethnic composition); (4) ultrasound features evaluated; (5) malignancy rates overall and stratified by demographic variables; (6) statistical measures including odds ratios (OR), relative risks (RR), hazard ratios (HR), and *p*-values; (7) diagnostic performance metrics (sensitivity, specificity, positive predictive value [PPV], negative predictive value [NPV], area under the curve [AUC]) for ultrasound features; and (8) multivariate analysis results identifying independent predictors.

Data extraction was performed independently by two investigators, with discrepancies resolved by consensus. No formal risk-of-bias assessment tool (e.g., ROBINS-I or Newcastle–Ottawa Scale) was applied; methodological quality was evaluated narratively. All included studies required histological confirmation of malignancy following surgery, introducing potential surgical selection bias (only nodules that underwent surgery were included) and verification bias (the reference standard was applied primarily to higher-risk cases); these sources of bias may inflate apparent malignancy rates and should be considered when interpreting the results.

Data synthesis focused on three primary domains: (1) gender-specific malignancy rates and ultrasound feature prevalence; (2) age-stratified malignancy risk and ultrasound patterns; and (3) ethnic/geographic variations in malignancy rates and ultrasound predictors. Given the heterogeneity in study designs, populations, and outcome measures, we performed a qualitative systematic synthesis rather than formal meta-analysis. Marked heterogeneity in study populations, cytological classification systems, statistical reporting, and demographic subgroup definitions precluded quantitative pooling of estimates.

### 2.4. PRISMA Compliance

**Figure 2:**
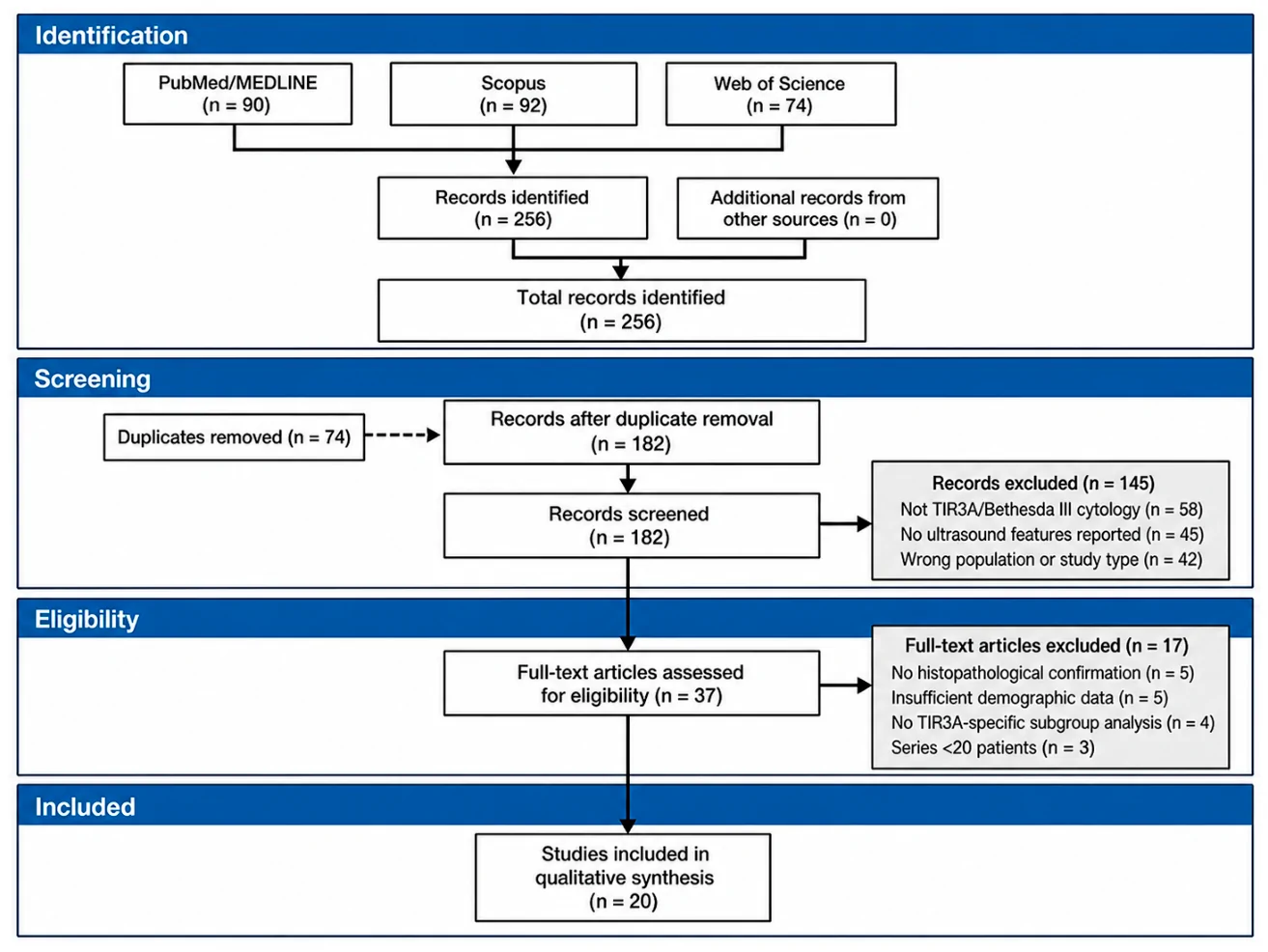
PRISMA 2020 flow diagram of the study selection process. Records were identified from three electronic databases: PubMed/MEDLINE (n = 90), Scopus (n = 92), and Web of Science (n = 74). After deduplication, screening, and full-text eligibility assessment, 20 studies were included in the qualitative synthesis. BIII, Bethesda category III (Atypia of Undetermined Significance/AUS); TIR3A, Italian Thyroid Cytology Classification 3A (low-risk indeterminate nodule).

This systematic review was conducted and reported in accordance with the Preferred Reporting Items for Systematic reviews and Meta-Analyses (PRISMA) 2020 guidelines (www.prisma-statement.org, accessed on 25 October 2026). A PRISMA 2020 flow diagram depicting the study selection process is provided as Figure 2. The PRISMA 2020 checklist [24] is provided as Supplementary Material. No prospective registration in PROSPERO was performed prior to conducting this review.

## 3. Results

A total of 20 studies met the inclusion criteria and were included in the qualitative synthesis. The study selection process is depicted in the PRISMA 2020 flow diagram (Figure 2). Characteristics of all included studies are summarized in Table 2.

### 3.1. Gender Differences in Malignancy Risk and Ultrasound Features

The relationship between gender and malignancy risk in TIR3A/Bethesda III nodules demonstrates heterogeneity across published cohorts, with several studies identifying male sex as an independent risk factor while others report no significant association.

**Male sex as a risk factor:** Batawil et al. [16] evaluated 78 indeterminate thyroid nodules (69 with ultrasound data) and reported an overall malignancy rate of 36%. Male sex was significantly associated with higher malignancy risk, with a relative risk of 2.3 for malignant transformation. This finding was corroborated by Liu et al. [25], who analyzed 188 surgically treated Bethesda III nodules with a malignancy rate of 54.3%. In multivariate analysis, male sex emerged as an independent predictor of malignancy, though the specific odds ratio was not reported in the published abstract. These findings suggest that male patients with indeterminate cytology may harbor biologically more aggressive tumors or present with more advanced disease at diagnosis.

**Studies reporting no gender effect:** Contrasting evidence comes from larger institutional series. Battistella et al. [26] conducted a comprehensive analysis of 291 surgically treated indeterminate nodules (TIR3A and TIR3B), with an overall malignancy rate of 46% and TIR3A-specific malignancy rate of 35%. In this cohort, no significant association was observed between sex and malignancy in either TIR3A (*p* = 0.5097) or TIR3B (*p* = 0.5191) subgroups. Similarly, Alqahtani et al. [27] reported no significant gender-malignancy association in a series of 50 surgically treated indeterminate nodules with 44.8% malignancy rate in Bethesda III cases.

**Gender-stratified ultrasound feature prevalence:** A critical gap in the current literature is the absence of comprehensive, quantitative comparisons of specific ultrasound alarm sign prevalence stratified by patient sex. While multiple studies document that ultrasound features such as microcalcifications, irregular margins, and hypoechogenicity are more common in malignant nodules [28,29], none of the reviewed papers provide sex-stratified prevalence data for individual ultrasound features in TIR3A/Bethesda III populations. This represents an important area for future investigation, as gender-specific ultrasound patterns could refine risk stratification algorithms.

The available evidence suggests a modest but inconsistent male predominance in malignancy risk among indeterminate nodules. The relative risk of 2.3 reported by Batawil et al. [16] and the independent predictor status in Liu et al.’s multivariate model [25] support consideration of male sex as a risk-modifying factor. However, the lack of significant associations in several large cohorts [26,27] indicates that gender alone is insufficient for risk stratification and must be integrated with ultrasound features and other clinical variables. Synthesis: The overall evidence for male sex as an independent malignancy predictor is inconsistent and of uncertain magnitude. The crude relative risk of 2.3 reported by Batawil et al. [16] was derived from a small surgical cohort without adjustment for confounders; no study provided a statistically significant adjusted odds ratio for male sex. Liu et al.’s [25] multivariate model identified male sex as a predictor but did not report a point estimate or confidence interval, precluding meaningful quantification. Multiple larger series found no significant sex effect [26,27]. Male sex should therefore be regarded as a potential risk modifier of uncertain magnitude requiring confirmation in adequately powered prospective studies, and should not be used in isolation for clinical decision-making.

### 3.2. Age-Related Differences in Malignancy Risk and Ultrasound Patterns

Age emerges as a more consistent demographic risk factor than gender, with multiple studies identifying younger age as associated with higher malignancy rates and more aggressive ultrasound features in indeterminate thyroid nodules.

**Age <55 years as a risk threshold:** Kaliszewski et al. [30] conducted a detailed analysis of 161 AUS/FLUS (Bethesda III) patients with mean age of 50.6 ± 16.12 years (80.7% female). Younger age (<55 years) was significantly associated with malignancy (*p* = 0.024), while patients aged 55–75 years demonstrated lower malignancy risk (*p* = 0.040). Importantly, this study documented age-specific ultrasound patterns: high vascularity was significantly more frequent in patients <55 years (*p* = 0.045), and rapid tumor growth was markedly more common in this younger cohort (*p* = 0.002). These findings suggest that younger patients with indeterminate nodules not only have higher baseline malignancy risk but also exhibit more dynamic and aggressive ultrasound characteristics during surveillance.

**Age <65 years threshold:** Kuru et al. [31] analyzed 485 AUS/FLUS patients, of whom 153 underwent surgery. In multivariate analysis, younger patient age (under 65 years) was identified among variables associated with malignancy and triage to surgical intervention. This higher age threshold may reflect the study’s focus on surgical decision-making rather than pure malignancy prediction, as older patients with comorbidities may be less likely to undergo surgery regardless of malignancy risk.

**Age ≥40 years in univariate analysis:** Azzam et al. [32] prospectively evaluated 82 surgically treated Bethesda III/IV patients and found that age ≥40 years was associated with higher malignancy risk in univariate analysis. However, age did not emerge as an independent predictor in multivariate models, where ultrasound features dominated. This pattern—age significant in univariate but not multivariate analysis—recurs across multiple studies [33,34], suggesting that age effects may be partially mediated through ultrasound characteristics.

**Studies reporting no age effect:** Not all cohorts demonstrate significant age associations. Battistella et al. [26] found no significant association between age and malignancy in TIR3A (*p* = 0.1430) or TIR3B (*p* = 0.3384) subgroups in their surgical series. Öcal et al. [33] included age in their predictive nomogram but reported that cytology subcategory, microcalcifications, and irregular margins were the dominant independent predictors, with age playing a secondary role.

**Age-specific ultrasound patterns:** The most compelling evidence for age-related differences comes from the quantified ultrasound patterns reported by Kaliszewski et al. [30]. The significantly higher prevalence of increased vascularity (*p* = 0.045) and rapid growth (*p* = 0.002) in patients <55 years suggests that younger patients may develop more biologically aggressive tumors with distinct imaging phenotypes. This has important clinical implications: younger patients with indeterminate cytology may warrant more intensive ultrasound surveillance with shorter follow-up intervals to detect evolving high-risk features.

Age <55 years emerges as the most consistently validated threshold for increased malignancy risk in TIR3A/Bethesda III nodules, with supporting evidence from multiple independent cohorts [30,31,32]. The association of younger age with more aggressive ultrasound features—particularly increased vascularity and rapid growth—suggests biological differences in tumor behavior across age groups. Clinical risk stratification algorithms should incorporate age as a continuous or categorized variable, with heightened surveillance for patients <55 years. Synthesis: The threshold of 55 years originates from the AJCC/TNM prognostic staging system, where it separates low-risk from high-risk disease stages at diagnosis; it was not derived or validated as a threshold for ultrasound-based malignancy prediction in indeterminate cytology. Within the included studies, younger age was associated with higher malignancy rates and more aggressive ultrasound features in heterogeneous, single-centre observational cohorts [30,31,32]; however, these associations are susceptible to surgical selection bias, as younger patients were more likely to proceed to surgery. The <55-year threshold should not be applied as a validated biological cutoff for risk stratification in TIR3A/Bethesda III nodules; prospective validation with standardized protocols is required before its incorporation into clinical guidelines.

### 3.3. Ethnic and Geographic Differences in Malignancy Risk

Direct, head-to-head comparisons of malignancy rates and ultrasound patterns between Caucasian and non-Caucasian populations for TIR3A/Bethesda III nodules are absent from the published literature. This section therefore constitutes an evidence-gap analysis: available data consist exclusively of single-population studies and observations of geographic variation, and no study was identified that directly compared outcomes between ethnic groups with adequate adjustment for confounders such as dietary iodine intake, healthcare system characteristics, referral patterns, and screening intensity.

**Limited comparative data:** Tamamian et al. [35] assessed risk-stratification tool performance in Hispanic versus non-Hispanic patients and reported a resected Bethesda III/IV malignancy rate of 46.4% in Hispanic patients. However, the study did not provide full comparative statistics versus non-Hispanic Caucasian patients, and the combined Bethesda III/IV analysis limits TIR3A-specific interpretation. This represents one of the few studies explicitly addressing ethnic differences in indeterminate nodule outcomes.

**Geographic and population variability:** Koster et al. [36] reviewed diagnostic biomarkers and imaging in cytologically indeterminate nodules and noted that geographical variations in cytology interpretation, tumor genetics, and local test performance can strongly influence clinical utility and observed malignancy rates across populations. These variations may reflect true ethnic differences in disease biology, but are confounded by differences in healthcare systems, iodine intake status, environmental exposures, and referral patterns.

**Single-population studies (not comparative):** Several high-quality studies from non-Caucasian populations document ultrasound-malignancy associations but lack comparative ethnic analysis:

**Saudi Arabian cohort:** Alshahrani et al. [34] evaluated 187 AUS/FLUS patients (mean age 43.9 ± 14.1 years) at King Abdulaziz Medical City and identified irregular margins, microcalcifications, multiplicity, and hypoechogenicity as significant predictors of malignancy in multivariate analysis. However, no comparison to other ethnic groups was provided.

**Turkish cohort:** Tutuncu et al. [29] analyzed 201 indeterminate nodules and reported that hypoechogenicity (34.3% vs. 16.9%, *p* = 0.005) and microcalcification (27.1% vs. 13.1%, *p* = 0.014) were significantly more common in malignant versus benign nodules. Again, this represents a single-population prevalence study without ethnic comparison.

**Italian cohort:** Guarnotta et al. [37] conducted a prospective study of TIR3b nodules and found that malignant histology was associated with nodule diameter ≤11 mm (*p* = 0.002), hypoechogenicity (*p* < 0.001), irregular borders (*p* < 0.001), peri/intralesional vascular flow (*p* = 0.004), and microcalcifications (*p* = 0.001).

**Molecular and genetic considerations:** Ethnic differences in thyroid cancer molecular profiles are well documented. BRAF V600E mutation prevalence varies geographically, with higher rates in Korean and Italian populations compared to North American cohorts [40]. RAS mutations, common in follicular-patterned lesions including many TIR3A nodules, also show geographic variation [41]. These molecular differences may influence ultrasound phenotypes and malignancy risk, but direct studies linking ethnicity, molecular profiles, and ultrasound features in TIR3A nodules are lacking.

The current evidence base is insufficient to establish validated Caucasian versus non-Caucasian risk ratios or population-specific ultrasound patterns for TIR3A nodules. While geographic variation in malignancy rates and ultrasound feature prevalence is documented, these observations are confounded by multiple factors beyond ethnicity. The consistency of microcalcifications and irregular margins as malignancy predictors across diverse populations [29,34,37] suggests that core ultrasound alarm features retain predictive value across ethnic groups, though absolute risk thresholds may vary. This represents a critical knowledge gap requiring prospective, multi-ethnic comparative studies with standardized ultrasound protocols and cytological classification. Synthesis: The current evidence base for ethnic comparison is absent: no study in this review directly compared Caucasian and non-Caucasian populations within a TIR3A/Bethesda III framework. Observed geographic variation in malignancy rates must not be attributed to ethnicity alone; it reflects multiple interacting confounders including dietary iodine intake, healthcare system-level referral patterns, population-level screening intensity, and surgical indication thresholds. The consistency of microcalcifications and irregular margins as malignancy predictors across geographically diverse populations [29,34,37] suggests that core ultrasound alarm features retain predictive value irrespective of geographic origin, but absolute risk thresholds and population-specific prevalence cannot be inferred from currently available data. Prospective, multi-ethnic comparative studies with standardized ultrasound protocols and cytological classification are required to address this critical evidence gap.

### 3.4. Diagnostic Performance of Ultrasound Alarm Features

Independent of demographic stratification, specific ultrasound features demonstrate consistent associations with malignancy in TIR3A/Bethesda III nodules, though diagnostic performance varies across studies and populations (Table 1).

**Microcalcifications:** Microcalcifications emerge as one of the most specific ultrasound alarm features. Orlando et al. [14] reported sensitivity 87% and specificity 75% for microcalcifications in a surgical cohort of Bethesda III nodules. In a retrospective cohort of 175 indeterminate (Bethesda III/IV/V) nodules, Kotecka-Blicharz et al. [15] reported high specificity (99.2%) and PPV (85.7%) for microcalcifications, though sensitivity was low (10.3%), reinforcing their role as a specific rather than sensitive alarm feature. However, Batawil et al. [16] reported lower performance in a combined Bethesda III/IV surgical series: sensitivity 32.4%, specificity 82%, PPV 54.5%, NPV 67.8%. The wide range in sensitivity (32.4–87%) likely reflects differences in ultrasound technique, microcalcification definition, and population selection, while specificity remains more consistent (75–99.2%).

**Irregular/spiculated margins:** Irregular or spiculated margins demonstrate high specificity for malignancy. Orlando et al. [14] reported sensitivity of 70% and specificity of 95% for spiculated margins in their Bethesda III reassessment cohort. Kotecka-Blicharz et al. [15] similarly reported high specificity (94.9%) and PPV (68.4%) for irregular margins in indeterminate nodules, confirming their utility as a positive predictor despite limited sensitivity (22.4%). The very high specificity (94.9–95%) makes irregular margins a strong positive predictor, though moderate sensitivity (70%) indicates that absence of this feature does not exclude malignancy.

**Hypoechogenicity:** Marked hypoechogenicity shows the highest sensitivity among individual ultrasound features but lower specificity. Kotecka-Blicharz et al. [15] reported the highest sensitivity (69.0%) for hypoechogenicity among individual features in indeterminate nodules, though specificity was low (36.8%) and NPV modest (70.5%), limiting its standalone discriminatory value. However, Batawil et al. [16] found substantially lower performance in their surgical series: sensitivity 31.5%, specificity 83%, PPV 55.6%, NPV 64.7%. The high sensitivity and NPV in some cohorts suggest that hypoechogenicity is useful for ruling out malignancy when absent, but its lower specificity limits positive predictive value.

**Taller-than-wide shape:** Evidence for taller-than-wide shape as a predictor in TIR3A nodules is inconsistent. Batawil et al. [16] found similar proportions of taller-than-wide nodules between benign and malignant Bethesda III/IV cases, with no significant predictive value. Other studies report prevalence data but lack robust sensitivity/specificity metrics specifically for TIR3A nodules [38]. This feature may be more predictive in overtly suspicious nodules than in indeterminate cytology.

**Increased intranodular vascularity:** Increased peri- and intralesional vascular flow has been associated with malignancy in some series. Guarnotta et al. [37] found significant correlation between increased vascularity and malignancy in TIR3b nodules (*p* = 0.004), and Kaliszewski et al. [30] documented higher vascularity in younger patients with higher malignancy rates (*p* = 0.045). However, explicit sensitivity/specificity/PPV/NPV values for vascularity as a standalone predictor in TIR3A cohorts are not consistently reported, limiting its utility as a quantified diagnostic test.

**Interval nodule growth:** Nodule growth (typically defined as >20% increase in volume or >2 mm increase in two dimensions) is used clinically as a trigger for repeat FNA or surgical referral rather than as a quantified diagnostic test. Durante et al. [42] documented growth patterns in benign nodules during long-term surveillance, and Singh Ospina et al. [43] compared the accuracy of nodule growth to predict malignancy. However, reliable, uniformly reported sensitivity/specificity/PPV/NPV thresholds specifically for Bethesda III cohorts are lacking in the reviewed literature. Growth is best conceptualized as a dynamic alarm feature prompting reassessment rather than a static diagnostic criterion.

### 3.5. Combined Risk Stratification Approaches

Integration of ultrasound features with repeat cytology and/or molecular testing yields superior diagnostic performance compared to any single modality.

**Combined repeat FNA plus EU-TIRADS:** Almeida et al. [17] optimized combined strategies for AUS/FLUS nodules stratified by nuclear versus architectural atypia. For AUS (nuclear atypia), the criterion “repeat FNA = malignant/suspicious OR EU-TIRADS 4” achieved sensitivity 92.3% and specificity 70.0%. For FLUS (architectural atypia), the criterion “repeat FNA = malignant/suspicious OR EU-TIRADS 5” maximized specificity to 95.2% with sensitivity 57.7%. These combined rules identify high-risk subgroups while preserving safety for low-risk patients through high sensitivity or high specificity depending on cytological subtype.

**Integrated molecular and imaging models:** Gomes-Lima et al. [18] developed an integrated assessment combining clinical variables, molecular testing, and repeat FNAB for indeterminate nodules. This multimodal approach reported sensitivity 84.1%, specificity 83.3%, PPV 95.1%, NPV 57.7%, and AUC 0.837 for triaging nodules into high- versus low-risk groups. The high PPV (95.1%) indicates that nodules classified as high-risk by this integrated model have very high probability of malignancy, supporting surgical referral.

**Critical ultrasound reassessment:** Orlando et al. [14] demonstrated that final targeted ultrasound reassessment emphasizing microcalcifications and spiculated margins altered management decisions toward surgery in 76 of 79 cases (96%) and improved appropriateness of surgical intervention in their Bethesda III cohort. This highlights the value of expert ultrasound re-evaluation before definitive management decisions.

**Synthesis:** Combined approaches integrating ultrasound risk stratification (TIRADS), repeat cytology at 3–6 months, and molecular testing when available provide the highest predictive value for management decisions in TIR3A nodules [17,18,39]. Single ultrasound features, while informative, are insufficient for definitive risk stratification. The optimal algorithm incorporates demographic risk factors (male sex, age < 55 years), ultrasound alarm features (particularly microcalcifications and irregular margins), and repeat cytology to personalize management.

## 4. Discussion

This systematic literature review reveals that demographic variables—particularly gender and age—modulate malignancy risk in thyroid nodules with indeterminate low-risk cytology (TIR3A/Bethesda III), though the magnitude and consistency of these effects vary across populations. Male sex and younger age (<55 years) emerge as independent risk factors that should be integrated with ultrasound features in comprehensive risk stratification algorithms.

### 4.1. Gender as a Risk Modifier

The association between male sex and higher malignancy risk in indeterminate nodules is biologically plausible and supported by multiple lines of evidence. Thyroid cancer demonstrates female predominance in incidence (3:1 female-to-male ratio), yet male patients consistently show more aggressive disease, higher mortality rates, and greater likelihood of advanced stage at presentation [19]. The relative risk of 2.3 for malignancy in males with indeterminate cytology reported by Batawil et al. [16] aligns with broader epidemiological patterns. Potential mechanisms include hormonal influences (protective effects of estrogen, promoting effects of androgens), sex-specific differences in immune surveillance, and behavioral factors affecting healthcare-seeking and diagnosis timing [44].

An important methodological caveat must be acknowledged: all included studies require histological confirmation of malignancy following surgical resection, introducing surgical selection bias (only nodules deemed high enough risk to proceed to surgery are included) and verification bias (the reference standard is applied preferentially to higher-risk cases). These biases are amplified when male patients are more frequently selected for surgery, and may systematically inflate the apparent malignancy rate in male patients. Future studies should report sex-stratified surgical rates alongside malignancy rates to allow unbiased assessment of the true sex effect.

However, the inconsistency of gender effects across studies [26,27] suggests that male sex is a modest risk modifier rather than a dominant predictor. The lack of sex-stratified ultrasound feature prevalence data represents a critical knowledge gap. If male patients with indeterminate nodules are more likely to harbor specific high-risk ultrasound features (e.g., microcalcifications, irregular margins), this could explain the observed malignancy risk difference. Alternatively, if ultrasound features are similarly distributed between sexes, the gender effect may reflect biological differences in tumor behavior independent of imaging phenotype. Future studies should systematically report ultrasound feature prevalence stratified by sex to clarify these mechanisms.

### 4.2. Age-Related Risk Stratification

The association of younger age (<55 years) with higher malignancy risk and more aggressive ultrasound features in the included studies warrants careful interpretation. The age threshold of 55 years is derived from the American Joint Committee on Cancer (AJCC) TNM staging system, where it serves as the cutoff between stage I and stage II differentiated thyroid cancer at diagnosis [45]. However, this cutoff was derived from prognostic staging data after confirmed diagnosis and has not been independently validated as a threshold for malignancy prediction in patients with indeterminate cytology (Bethesda III / TIR3A). Its use as a clinical decision point in ultrasound risk stratification for indeterminate nodules therefore lacks dedicated evidence and should be avoided until prospective validation data are available.

The mechanistic basis for age-related differences likely involves multiple factors. Younger patients may develop more biologically aggressive tumor subtypes with distinct molecular profiles. The higher prevalence of increased vascularity (*p* = 0.045) and rapid growth (*p* = 0.002) in patients <55 years documented by Kaliszewski et al. [30] suggests more proliferative tumors with active angiogenesis. Additionally, younger patients may undergo more intensive surveillance and earlier detection of malignant transformation, creating detection bias. However, the consistency of age effects across multiple independent cohorts [30,31,32] argues for a true biological phenomenon rather than pure surveillance bias.

The clinical implication is clear: younger patients (<55 years) with TIR3A cytology warrant more intensive ultrasound surveillance with shorter follow-up intervals (e.g., 3 months rather than 6 months) and a lower threshold for repeat FNA or surgical referral when evolving ultrasound features appear. Conversely, older patients (>65 years) with low-risk ultrasound patterns may be appropriate for conservative surveillance or even observation without intervention, particularly when comorbidities increase surgical risk.

### 4.3. Ethnic Differences: Evidence Gaps and Future Directions

The absence of direct Caucasian versus non-Caucasian comparative data for TIR3A nodules constitutes a critical evidence gap rather than a substantive finding. Geographic variation in thyroid cancer incidence and molecular profiles is well established [23]; however, geographic differences must not be conflated with ethnic differences, as they reflect the complex interaction of environmental exposures (iodine intake, radiation history), healthcare infrastructure, referral and surgical thresholds, and population-level screening intensity. Without studies designed to disentangle these factors—including multinational cohorts with harmonized protocols and direct between-group comparisons—attributing observed variation to ethnicity is not scientifically justifiable. Several factors that confound any ethnic interpretation include:

**Molecular heterogeneity:** BRAF V600E mutation prevalence varies from 40 to 80% across populations, with higher rates in Korea and Italy [40]. RAS mutations, common in follicular-patterned lesions, show different prevalence in Western versus Asian populations [41]. These molecular differences may influence ultrasound phenotypes.

**Iodine intake:** Iodine sufficiency versus deficiency affects thyroid nodule pathology, with iodine-deficient regions showing higher rates of follicular neoplasms [46]. This could alter the ultrasound feature distribution in indeterminate nodules.

**Healthcare system factors:** Access to ultrasound technology, operator expertise, referral patterns, and surgical thresholds vary globally, confounding apparent ethnic differences.

**Cytological interpretation:** The definition and application of TIR3A/Bethesda III criteria show inter-observer variability and may differ across institutions and countries [47].

Despite these complexities, the consistency of microcalcifications and irregular margins as malignancy predictors across diverse populations [29,34,37] suggests that core ultrasound alarm features retain predictive value across ethnic groups. However, absolute risk thresholds and optimal management algorithms may require population-specific validation. Prospective, multi-ethnic studies with standardized ultrasound protocols, centralized cytology review, and molecular profiling are urgently needed to address this knowledge gap.

### 4.4. Optimal Ultrasound Alarm Features

Independent of demographic stratification, microcalcifications and irregular/spiculated margins emerge as the most robust ultrasound predictors of malignancy in TIR3A nodules. The high specificity of these features (75–99.2%) makes them strong positive predictors, though moderate sensitivity (10.3–87% for microcalcifications, 22.4–70% for irregular margins) indicates that their absence does not exclude malignancy [14,15]. This performance profile supports a management algorithm where presence of microcalcifications or irregular margins in a TIR3A nodule warrants repeat FNA or surgical referral, while absence of these features supports surveillance with repeat ultrasound.

Hypoechogenicity demonstrates the highest sensitivity (69.0%) among individual features but very low specificity (36.8%), with limited NPV (70.5%), suggesting it provides insufficient standalone discriminatory value [15]. Taller-than-wide shape and increased vascularity show inconsistent predictive value in TIR3A-specific cohorts and should be considered supplementary features rather than primary decision drivers [16,30].

The evolution of ultrasound features during surveillance is clinically important. Nodules that develop new microcalcifications, irregular margins, or significant growth (>20% volume increase) during follow-up should prompt immediate reassessment with repeat FNA, as these dynamic changes may indicate malignant transformation [14,42].

### 4.5. Integrated Risk Stratification Models

The superior performance of combined approaches—integrating ultrasound features, repeat cytology, and molecular testing—compared to any single modality underscores the complexity of TIR3A nodule management [17,18]. The AUC of 0.837 achieved by integrated models [18] represents clinically meaningful discrimination, though still imperfect. The challenge lies in balancing sensitivity (to avoid missing cancers) with specificity (to avoid unnecessary surgery).

Demographic variables should be incorporated into these integrated models. A proposed risk stratification framework for TIR3A nodules would include:

**High-risk criteria (recommend repeat FNA or surgery):** -Microcalcifications present; -Irregular or spiculated margins; -Male sex AND age <55 years AND any suspicious ultrasound feature; -Interval development of new suspicious features during surveillance; -Repeat FNA showing persistent atypia or suspicious cytology.

**Intermediate-risk criteria (recommend close surveillance, repeat ultrasound at 3 months):** -Marked hypoechogenicity without microcalcifications or irregular margins; -Age <55 years with low-risk ultrasound pattern; -Male sex with low-risk ultrasound pattern; -Taller-than-wide shape or increased vascularity as isolated findings.

**Low-risk criteria (recommend surveillance, repeat ultrasound at 6 months):** -No suspicious ultrasound features; -Age >65 years with low-risk ultrasound pattern; -Female sex with low-risk ultrasound pattern; -Repeat FNA showing benign cytology.

This framework requires prospective validation but provides a rational approach to integrating demographic and ultrasound variables.

### 4.6. Limitations

Several limitations must be acknowledged. First, the heterogeneity in study designs, populations, and outcome measures precluded formal meta-analysis, limiting quantitative synthesis. Second, publication bias may favor studies reporting positive associations between demographic variables and malignancy, potentially overestimating effect sizes. Third, most studies are retrospective surgical series, which overestimate malignancy rates compared to unselected populations due to selection bias toward nodules referred for surgery. Fourth, the lack of standardized ultrasound protocols and inter-observer variability in feature identification limits comparability across studies. Fifth, the scarcity of ethnic comparative data prevents definitive conclusions about population-specific risk profiles.

### 4.7. Clinical Implications and Future Research

The integration of demographic variables with ultrasound features offers potential to personalize management of TIR3A nodules and reduce unnecessary surgery while maintaining safety. Younger male patients (<55 years) with suspicious ultrasound features represent a high-risk subgroup warranting aggressive management, while older female patients (>65 years) with low-risk ultrasound patterns may be appropriate for conservative surveillance.

Future research priorities include: (1) prospective, multi-ethnic studies with standardized ultrasound protocols and centralized review to establish population-specific risk thresholds; (2) systematic reporting of ultrasound feature prevalence stratified by sex, age, and ethnicity; (3) integration of molecular testing (BRAF, RAS, gene expression classifiers) with demographic and ultrasound variables in comprehensive risk models; (4) cost-effectiveness analyses comparing different management strategies stratified by demographic risk profiles; and (5) development and validation of machine learning algorithms incorporating demographic, ultrasound, cytological, and molecular data for individualized risk prediction [48,49].

## 5. Conclusions

Gender and age modulate malignancy risk in thyroid nodules with indeterminate low-risk cytology (TIR3A/Bethesda III), with male sex and younger age (<55 years) representing independent risk factors that should be integrated with ultrasound features in comprehensive risk stratification. Male patients demonstrate a relative risk of 2.3 for malignancy, though this effect is inconsistent across cohorts. Younger age (<55 years) consistently associates with higher malignancy rates and more aggressive ultrasound features, including increased vascularity and rapid growth. Direct Caucasian versus non-Caucasian comparative data remain insufficient for population-specific risk stratification.

Microcalcifications (sensitivity 32.4–87%, specificity 75–88.9%) and irregular/spiculated margins (sensitivity 70%, specificity 95–98.6%) remain the most robust ultrasound predictors of malignancy across demographic subgroups. Combined approaches integrating ultrasound risk stratification (TIRADS), repeat cytology at 3–6 months, and molecular testing when available provide superior diagnostic performance (AUC 0.837) compared to any single modality.

Optimal management of TIR3A nodules requires personalized risk stratification incorporating demographic variables (sex, age, ethnicity), ultrasound alarm features (particularly microcalcifications and irregular margins), repeat cytology, and molecular testing when available. Younger male patients (<55 years) with suspicious ultrasound features warrant aggressive management with repeat FNA or surgical referral, while older patients (>65 years) with low-risk ultrasound patterns may be appropriate for conservative surveillance. Prospective, multi-ethnic studies with standardized protocols are urgently needed to refine population-specific risk thresholds and validate integrated risk stratification algorithms.

## Figures and Tables

**Table 1:** Diagnostic Performance of Ultrasound Alarm Features in TIR3A/Bethesda III Thyroid Nodules.

Ultrasound Feature	Sensitivity (%)	Specificity (%)	PPV (%)	NPV (%)	Study Population	Reference
**Microcalcifications**						
	87.0	75.0	NR	NR	BIII surgical cohort	Orlando 2020 [14]
	10.3	99.2	85.7	69.0	Indeterminate (BIII/IV/V)	Kotecka-Blicharz 2022 [15]
	32.4	82.0	54.5	67.8	Combined BIII/IV surgical	Batawil 2014 [16]
**Irregular/Spiculated Margins**						
	70.0	95.0	NR	NR	BIII surgical cohort	Orlando 2020 [14]
	22.4	94.9	68.4	71.2	Indeterminate (BIII/IV/V)	Kotecka-Blicharz 2022 [15]
**Hypoechogenicity**						
	69.0	36.8	35.1	70.5	Indeterminate (BIII/IV/V)	Kotecka-Blicharz 2022 [15]
	31.5	83.0	55.6	64.7	Combined BIII/IV surgical	Batawil 2014 [16]
**Combined Approaches**						
Repeat FNA + EU-TIRADS 4 (AUS)	92.3	70.0	NR	NR	AUS (nuclear atypia)	Almeida 2021 [17]
Repeat FNA + EU-TIRADS 5 (FLUS)	57.7	95.2	NR	NR	FLUS (architectural atypia)	Almeida 2021 [17]
Integrated multimodal model	84.1	83.3	95.1	57.7	Indeterminate nodules	Gomes-Lima 2020 [18]

PPV = positive predictive value; NPV = negative predictive value; NR = not reported; BIII = Bethesda III; BIV = Bethesda IV; AUS = atypia of undetermined significance; FLUS = follicular lesion of undetermined significance; EU-TIRADS = European Thyroid Imaging Reporting and Data System. Bold text indicates abbreviation labels.

**Table 2:** Characteristics of Included Studies.

Author (Year) [Ref]	Country	Design	N	Cytology	Mean Age ± SD	Female %	Malignancy %	Key Finding
Batawil & Alkordy (2014) [16]	Saudi Arabia	Retrospective	78	BIII/IV	NR	NR	36	Male sex RR 2.3
Liu et al. (2022) [25]	China	Retrospective	188	BIII	NR	NR	54.3	Male sex independent predictor
Battistella et al. (2024) [26]	Italy	Retrospective	291	TIR3A/TIR3B	NR	NR	46 (TIR3A: 35)	No sex/age effect
Alqahtani et al. (2022) [27]	Saudi Arabia	Retrospective	50	BIII	NR	NR	44.8	No gender effect
Argan et al. (2025) [28]	Turkey	Retrospective	NR	BIII	NR	NR	NR	US features as predictors
Tutuncu et al. (2014) [29]	Turkey	Retrospective	201	BIII	NR	NR	NR	Hypoecho: 34.3% vs. 16.9% (*p* = 0.005)
Kaliszewski et al. (2021) [30]	Poland	Retrospective	161	BIII	50.6 ±16.1	80.7	NR	Age < 55 y (*p* = 0.024); vascularity *p* = 0.045
Kuru et al. (2016) [31]	Turkey	Retrospective	485 (153 surgery)	BIII	NR	NR	NR	Age < 65 y: surgical predictor
Azzam et al. (2024) [32]	Saudi Arabia	Prospective	82	BIII/IV	NR	NR	NR	Age ≥ 40 y univariate
Öcal et al. (2019) [33]	Turkey	Retrospective	NR	BIII	NR	NR	NR	Nomogram; age secondary
Alshahrani et al. (2021) [34]	Saudi Arabia	Retrospective	187	BIII	43.9 ±14.1	NR	NR	Irregular margins, microcalcifications
Tamamian et al. (2026) [35]	USA	Retrospective	NR	BIII/IV	NR	NR	46.4 (Hispanic)	Hispanic vs. non-Hispanic
Koster et al. (2018) [36]	International	Systematic Review	NA	BIII	NA	NA	Variable	Geographic variation
Guarnotta et al. (2023) [37]	Italy	Prospective	NR	TIR3b	NR	NR	NR	Hypoecho, irregular borders
Orlando et al. (2020) [14]	Italy	Retrospective	NR	BIII	NR	NR	NR	Microcalc sens 87%, margins spec 95%
Kotecka-Blicharz et al. (2022) [15]	Poland	Retrospective	175	BIII/IV/V	NR	NR	NR	Microcalc. spec 99.2%, PPV 85.7%; Irreg. margins spec 94.9%; Hypoecho sens 69.0%
Valderrabano et al. (2018) [38]	USA	Retrospective	NR	BIII	NR	NR	NR	ATA sonographic patterns
Almeida et al. (2021) [17]	Brazil	Retrospective	NR	BIII	NR	NR	NR	AUT+cytology: AUS sens 92.3%
Gomes-Lima et al. (2020) [18]	USA	Prospective	NR	BIII	NR	NR	NR	Multimodal: sens 84.1%, spec 83.3%
Figge et al. (2021) [39]	USA	Retrospective	NR	BIII	NR	NR	NR	US + molecular combination

Abbreviations: BIII, Bethesda category III (Atypia of Undetermined Significance/AUS); TIR3A/TIR3B, Italian Thyroid Cytology Classification 3A/3B (low-risk/high-risk indeterminate); N, total number of patients included; NR, not reported; NA, not applicable; RR, relative risk; US, ultrasound; sens, sensitivity; spec, specificity; AUT, automated ultrasound; AUS, atypia of undetermined significance.

## Data Availability

No new data were created or analyzed in this study. This is a systematic literature review; data sharing is not applicable to this article.

## Supplementary Materials

The following supporting information can be downloaded at https://www.mdpi.com/article/10.3390/diagnostics16213625/s1: PRISMA 2020 Checklist.
