# Supplementary material for: Gender, Age, and Ethnic Differences in Ultrasound Predictors of Malignancy Risk in Thyroid Nodules with Indeterminate Low-Risk Cytology (TIR3A): A Systematic Literature Review
Source: Diagnostics (Basel). 2026 Nov 3;16(21):3625. doi: 10.3390/diagnostics16213625 (PMC13761595; doi:10.3390/diagnostics16213625)
Supplement: Supplementary file 1 [file diagnostics-16-03625-s001.zip › diagnostics-4625058-supplementary.pdf]

PRISMA 2020 Checklist

Preferred Reporting Items for Systematic Reviews and Meta-Analyses (PRISMA) 2020

Systematic review: Thyroid Nodule Risk Stratification: The Role of Patient Demographics and Ultrasound Features in Bethesda III/TIR3A Thyroid Nodules

Reference: Page MJ, McKenzie JE, Bossuyt PM, et al. The PRISMA 2020 statement: an updated guideline for reporting systematic reviews. BMJ. 2021;372:n71. doi: 10.1136/bmj.n71

Section & Topic	Item #	Checklist Item	Location in Manuscript
**TITLE**	1	Title: Identify the report as a systematic review.	*Title page*
**ABSTRACT**	2	Abstract: See the PRISMA 2020 for Abstracts checklist. Provide a structured summary including background, objectives, eligibility criteria, information sources, risk of bias, synthesis, results, limitations, conclusions, and systematic review registration number.	*Abstract*
**INTRODUCTION**	3	Rationale: Describe the rationale for the review in the context of existing knowledge.	*Section 1 (Introduction)*
	4	Objectives: Provide an explicit statement of the objective(s) or question(s) the review addresses.	*Section 1 (Introduction)*
**METHODS**	5	Eligibility criteria: Specify the inclusion and exclusion criteria for the review and how studies were grouped for the syntheses.	*Section 2.2*
	6	Information sources: Specify all databases, registers, websites, organisations, reference lists and other sources searched or consulted to identify studies. Specify the date when each source was last searched or consulted.	*Section 2.1*
	7	Search strategy: Present the full search strategies for all databases, registers and websites, including any filters and limits used.	*Section 2.1*
	8	Selection process: Specify the methods used to decide whether a study met the inclusion criteria of the review, including how many reviewers screened each record and each report retrieved, whether they worked independently, and if applicable, details of automation tools used.	*Section 2.2*
	9	Data collection process: Specify the methods used to collect data from reports, including how many reviewers collected data from each report, whether they worked independently, and any processes for obtaining or confirming data from study investigators.	*Section 2.3*

Section & Topic	Item #	Checklist Item	Location in Manuscript
	10	Data items: List and define all outcomes for which data were sought. Specify whether all results compatible with each outcome domain in each study were sought.	*Section 2.3*
	11	Study risk of bias assessment: Specify the methods used to assess risk of bias in the included studies, including details of the tool(s) used, how many reviewers assessed each study and whether they worked independently.	*Section 2.3*
	12	Effect measures: Specify for each outcome the effect measure(s) (e.g. odds ratio, mean difference) used in the synthesis or presentation of results.	*Section 2.3*
	13	Synthesis methods: Describe the methods used to tabulate or visually display results of individual studies and syntheses (if performed). For meta-analyses, describe methods for handling data and combining results from studies.	*Section 2.3; Section 2.4*
	14	Reporting bias assessment: Describe any methods used to assess risk of bias due to missing results in a synthesis (arising from reporting biases).	*Section 2.3*
	15	Certainty assessment: Describe any methods used to assess certainty (or confidence) in the body of evidence for an outcome.	*Section 2.3*
**RESULTS**	16	Study selection: Describe the results of the search and selection process, including results of searches of databases, registers, websites and other sources; number of records identified, screened, assessed for eligibility, and included in the review, with reasons for exclusions at each stage; preferably using a flow diagram.	*Section 3 (Introduction); Figure 2*
	17	Study characteristics: Cite each included study and present its characteristics.	*Table 2*
	18	Risk of bias in studies: Present assessments of risk of bias for each included study.	*Section 3; Table 1*
	19	Results of individual studies: For all outcomes, present, for each study: (a) summary statistics for each group (where appropriate) and (b) an effect estimate and its precision, ideally using structured tables or plots.	*Sections 3.1–3.3*
	20	Results of syntheses: For each synthesis, briefly summarise the characteristics and risk of bias among contributing studies. Present results of all statistical syntheses conducted. If meta-analysis was done, present for each the summary estimate and its precision.	*Sections 3.1–3.3*
	21	Reporting biases: Present assessments of risk of bias due to missing results (arising from reporting biases) for each synthesis assessed.	*Section 4 (Discussion)*

Section & Topic	Item #	Checklist Item	Location in Manuscript
	22	Certainty of evidence: Present assessments of certainty (or confidence) in the body of evidence for each outcome assessed.	*Section 4 (Discussion)*
**DISCUSSION**	23	Discussion: Provide a general interpretation of the results in the context of other evidence.	*Section 4 (Discussion)*
	24	Limitations of evidence: Discuss any limitations of the evidence included in the review.	*Section 4 (Discussion)*
	25	Limitations of review process: Discuss any limitations of the review process used.	*Section 4 (Discussion)*
	26	Conclusions: Provide a general interpretation of the results in the context of other evidence, and implications for future research.	*Section 5 (Conclusions)*
**OTHER INFORMATION**	27	Registration and protocol: Provide registration information for the review, including register name and registration number, or state that the review was not registered. Indicate if a review protocol exists and, if so, provide a link to the protocol.	*Section 2.4*
	28	Support: Describe sources of financial or other support for the review, and the role of the funders or sponsors in the review.	*Acknowledgments*
	29	Competing interests: Declare any competing interests of review authors.	*Author Contributions / Conflicts of Interest*

Note: This checklist was prepared as supplementary material in accordance with the MDPI journal requirements for systematic reviews submitted to Diagnostics. Items marked 'N/A' are not applicable to this qualitative synthesis, which did not perform formal meta-analysis.
